# Supplementary material for: The LIM Domain Protein BmFHL2 Inhibits Egg Production in Female Silkworm, Bombyx mori
Source: Cells. 2023 Jan 31;12(3):452. doi: 10.3390/cells12030452 (PMC9913792; doi:10.3390/cells12030452)
Supplement: Supplementary file 1 [file cells-12-00452-s001.zip › cells-2136413-supplementary.pdf]

The LIM domain protein BmFHL2 inhibits egg production in female silkworm, *Bombyx mori*

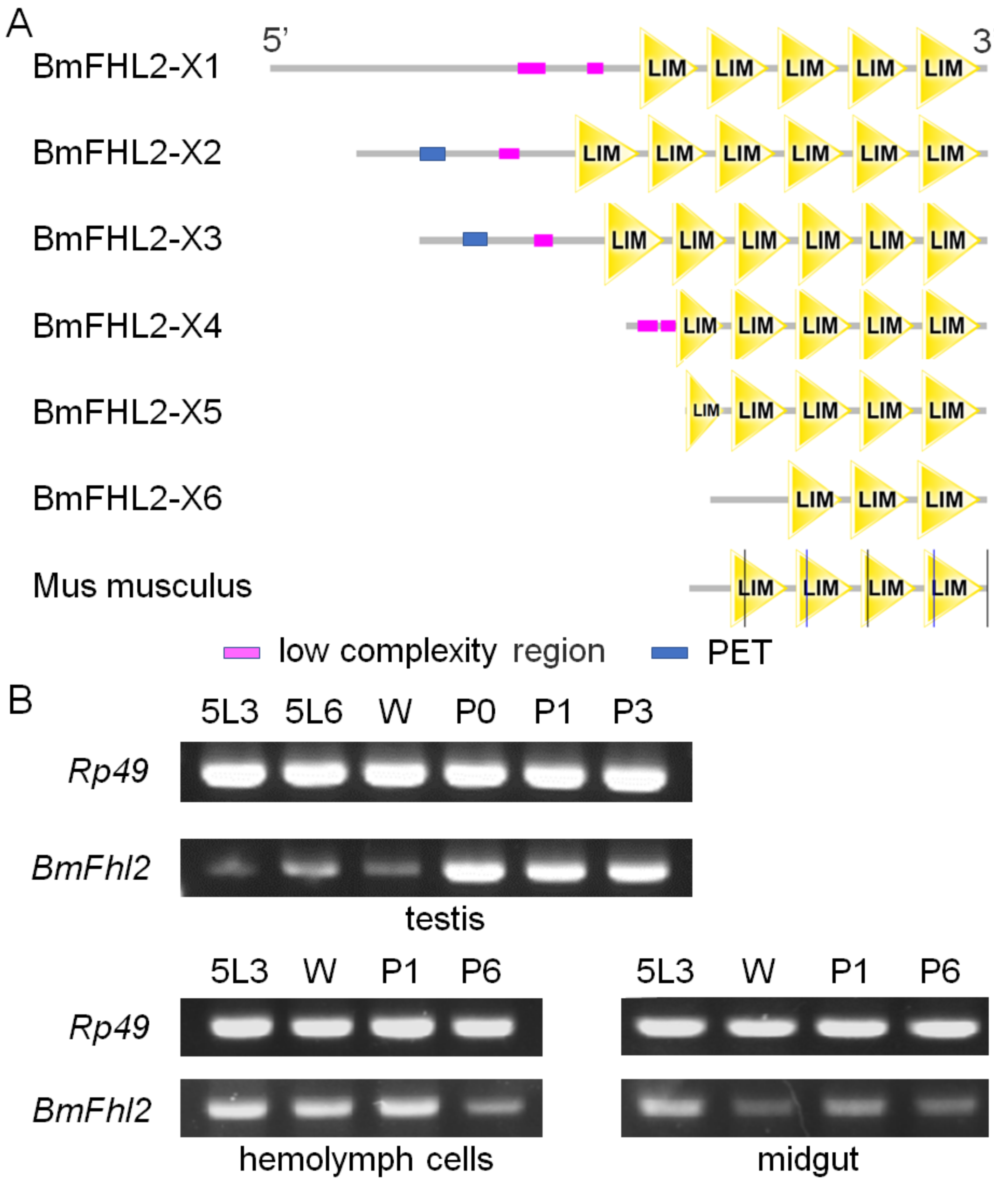

Figure S1. Schematic diagram of the domain analysis of BmFHL2. (**A**) Schematic diagram of the structure of different shears of silkworm FHL2 and mouse FHL2. (**B**) *BmFhl2* expression in testis, hemolymph cells and midgut at different time points. Total RNA was extracted from testis, hemolymph cells and midgut at different time points for RT-PCR analysis. *Rp49* was used as a reference gene. All samples were collected and mixed for three silkworm populations. 5L3: 5th instar 3 days, 5L6: 5th instar 6 days, W: wandering stage, P0: pupal stage 0 days, P1: pupal stage 1 day, P3: pupal stage 3 days, P6: pupal stage 6 days.

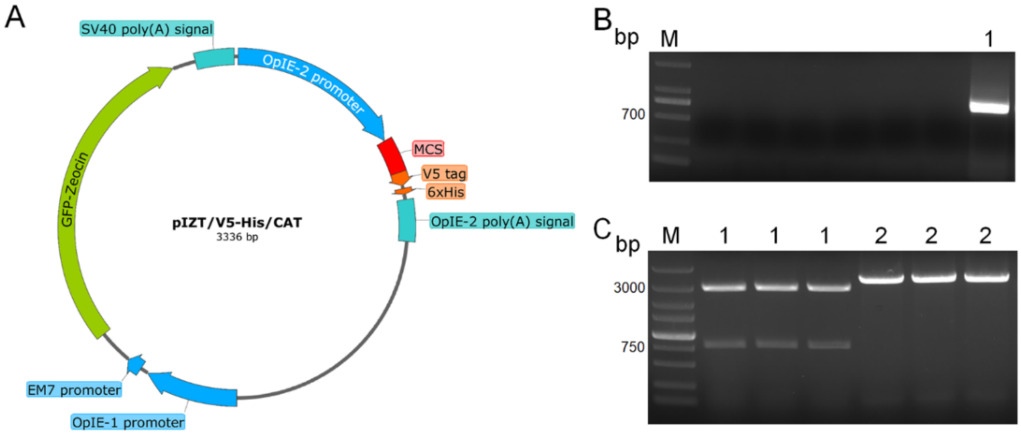

Figure S2. Overexpression of *BmFhl2* in BmN cells. (**A**) Schematic diagram of the *BmFhl2*.A.pIZT/V5-His plasmid overexpressed in BmN cells. (**B**) *BmFhl2-X6* was amplified from the cDNA of *B. mori* ovary at 5L3, and the product was subjected to agarose gel electrophoresis. M: Marker; 1: Target fragment. (**C**) Schematic diagram of electrophoresis of enzyme digestion products. M: Marker; 1: Not Ⅰ and Kpn Ⅰ enzyme digestion products of T vector; 2: Not Ⅰ and Kpn Ⅰ enzyme digestion products of empty vector.

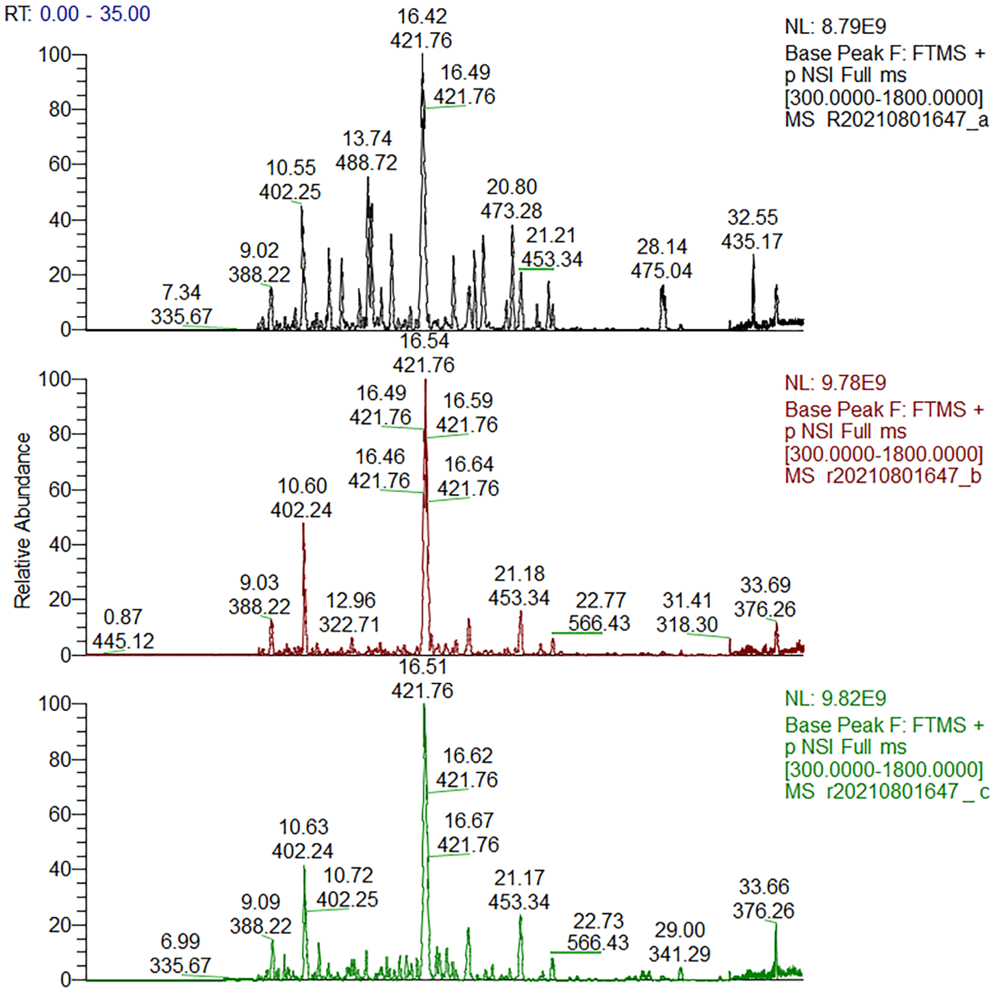

Figure S3. Co-IP mass spectrometry identification.

Table S1. Identification of interacting proteins of BmFHL2 by mass spectrometry.

NO.	Protein name	MW (KDa)	PepCount/ Unique Pepcount	Cover percent	sequence
a-1	26S proteasome non-ATPase regµLatory subunit 11	47.2	15/15	34.92%	TYKPELEEDAVVR
a-2	proliferation-associated protein 2G4	42.1	8/8	21.9%	VLEQVIAK
a-3	26S proteasome non-ATPase regµLatory subunit 13	43	7/7	20.52%	YVGCADGGEALP ATER
a-4	probable citrate synthase 2	51.8	8/8	18.49%	VVPPILTELGK
a-5	FAD-dependent oxidoreductase domain-containing protein 1	43.5	6/6	15.35%	IIDTENNVINAK
a-6	BTB/POZ domain-containing protein At1g21780	41.9	3/3	7.48%	EETIESQK
b-1	glutamate dehydrogenase	61.4	25/25	43.86%	IIAEAANGPTTPAA DK
b-2	ATP synthase subunit beta, mitochondrial	54.8	15/15	40.94%	FTQAGSEVSALLG R
b-3	low molecular lipoprotein 30K pBmHPC-12	29.2	3/2	14.84%	VVYGGNSADSTR
b-4	low molecular lipoprotein 30K pBmHPC-21	30.2	2/1	10.27%	VIFGTNTADTTR
b-5	low molecular lipoprotein 30K pBmHPC-19	27.4	2/2	6.33%	LIFAENAIK
c-1	polyubiquitin	17.2	3/3	39.22%	TITLEVEPSDTIENV K
c-2	ecdysteroid-phosphate phosphatase	161.1	2/2	1.4%	AELPLAALQR

Table S2. The sequence of primers.

Gene name	Primer name	Sequence (5′-3′)
BmRp49	Rp49-q-F	CAGGCGGTTCAAGGGTCAATAC
	Rp49-q-R	TACGGAATCCATTTGGGAGCAT

BmRp49	Rp49-RT-F	GACCTGTTTACAGGCCGACA
	Rp49-RT-R	ACTCTGATGCTGAGCTGCTG
BmFHL2	FHL2-q-F	TTCTTGCACCAAGCCCATCA
	FHL2-q-R	CCTGTCCGTCGGTGATGAAG
BmFHL2	FHL2-RT-F	TTCCGTGCTGGAACCAAGAA
	FHL2-RT-R	CGCAGATGAAGCAGTCGTTG
Vg	Vg-F	CTTGTGCCATCGATAGAACAG
	Vg-R	GTCGATATTGCATCCCCATC
30Kc19	30Kc19-F	GCGGTTGAAAAGAGCAAGCA
	30Kc19-R	ACTGGGAAACAATCCCGGAC
VgR	VgR-F	GAGTGCCTGGGCGAGGATGT
	VgR-R	CTGAGCGTCTGGCTTGTGA
EcR	EcR-F	GTGCTGGATGTGGTTGCTGAGGT
	EcR-R	CCGCTGGTCTAATAACGGTGGCT
USP	USP-F	ATGTCACCGCCCGAAATGAAGCC
	USP-R	CTGCTGGGATGAGCGTCTTCTGT
E75a	E75a-F	TCGGTCGAGCTTGAGTGAGA
	E75a-R	TAGCACAGGAGTGCATGAGC
siBmFHL2-405	F	CCGGCUGCUACGAGGACAATT
	R	UUGUCCUCGUAGCAGCCGGTT
siBmFHL2-777	F	GCGCCAAGCAGAAGCUCAUTT
	R	AUGAGCUUCUGCUUGGCGCTT
si NC		UUCUCCGAACGUGUCACGUTT
BmFHL2-X6	F	GGTACCATGTCAGCGGACGTCTTGAG（Kpn Ⅰ）
	R	GCGGCCGCTCATGAGCTTCTGCTTGGC（Not Ⅰ）
